# Epidemiological Study of *Cryptococcus gattii* Complex Infection in Domestic and Wild Animals in Oregon

**DOI:** 10.3390/vetsci12020185

**Published:** 2025-02-19

**Authors:** Sophia Ballard, Alexandria Montgomery, Ian Rose, Shawn Lockhart, Emilio DeBess, Luiz E. Bermudez

**Affiliations:** 1 Diagnostic Laboratory, Bacteriology and Parasitology Section, Carlson College of Veterinary Medicine, Corvallis, OR 97331, USA; sophia.ballard@oregonstate.edu (S.B.); montgoal@oregonstate.edu (A.M.); ian.rose@oregonstate.edu (I.R.); 2 Department of Microbiology, College of Science, Oregon State University, Corvallis, OR 97331, USA; 3 Center for Disease Control and Prevention, Atlanta, GA 30333, USA; shawn.lockhart@cdc.gov; 4 Oregon Health Authority, Portland, OR 97232, USA; emilio.debess@oha.oregon.gov; 5 Department of Biomedical Sciences, Carlson College of Veterinary Medicine, Oregon State University, Corvallis, OR 97331, USA

**Keywords:** *Cryptococcus gattii*, state of Oregon, epidemiology, genotypes, animal infection

## Abstract

The members of the *Cryptococcus gattii* species complex are the etiologic agents of potentially fatal human infection. *C. gattii* causes disease in both immunocompetent and immunocompromised hosts. In the early 2000s, infection caused by *C. gattii* emerged in the Pacific Northwest of the US. While many studies have been published about the human infection, the epidemiological characteristics of the infection in animals, with a possible role in human infection, have not been in investigated. Cases of *C. gattii* diagnosed in animals in Oregon from 2008 to 2019 were cataloged by county, species of animal, site of the infection, season of the year, and *C. gattii* genotype. One hundred and nine cases were diagnosed, and among the genotypes of *C. gattii,* VGII (*Cryptococcus deuterogatti*) with the genotypes VGIIa, VGIIb, and VGIIc was responsible for 98% of the cases. VGIIa was identified in more than 50% of the animals, and *Cryptococcus bacilliporus* (VGIII) was only isolated from cat patients. The majority of the infections were diagnosed in dogs and cats, although caprines, equines, camelids, ovines, and elk were also seen with the disease. The most common site of infection in dogs was the brain; that in cats was the nasal cavity and the skin, while the lung was the most affected site in caprines, equines, camelids and elk. Marion and Lane Counties account for the majority of the infections, followed by Clackamas, Benton, and Multnomah Counties. The infection was predominantly identified during the Fall and Winter months, except for Benton County, where it was seen more commonly during the Summer months. This study reviews all the cases identified by the Department of Public Health and by the veterinarians in Oregon in the years between 2008 and 2019.

## Simple Summary

An outbreak of infection caused by *Cryptococcus gattii* occurred in the Pacific Northwest region of the North America from 2008 to 2019. The pathogen is found in the environment and can infect humans and animals. This study is the first comprehensive study about the infection in domestic and wild animals. The study contains the incidence of infection by counties and months of the year, the genotypes of the pathogen causing those infections, the affected animal species, and the sites of infections in individual animals. The study looks at environmental factors that could have been associated with the infections. The study offers important information about the regions in the state, the clinical presentations, and the broad number of species of animals affected.

## 1. Introduction

*Cryptococcus gattii* is an emerging fungal pathogen associated with potentially severe disease in healthy individuals. *C. gattii* is distinct from *Cryptococcus neoformans*, known to commonly infect immunosuppressed individuals [1]. *C. gattii* is most prevalent in the Pacific Northwest region of North America, although it has been identified sporadically in other regions of the United States [2,3]. In Oregon, infections caused by *C. gattii* were first observed in 2004, and the infection was then subsequently evidenced in the State of Washington, suggesting a spread from British Columbia. Mortality in humans with the infection varies between 13 and 34% [1,2,3], although in animals it is unknown.

Cryptococcal diseases in animals are often characterized by upper respiratory symptoms, pneumonia, subcutaneous nodes, or central nervous system or ocular disorders. In British Columbia, the disease was recognized in wild and domestic animals before cases in humans were diagnosed. In Oregon, the infection caused by VGIIa was initially seen in one cat, two dogs, and two alpacas [4]. The outbreak was considered to be caused by migration of the pathogen after being carried by humans or animals (there is documentation of it being transported in tires of cars, [5]), followed by its spread into the environment. In Canada, *C. gatti* was recovered from acidic soil as well as from trees, and in Oregon, DeBess and colleagues identified the genotype VGIIa in trees that are typical of the Northwest region [6]. A study in 1990 from Australia reported that *C. gattii* was isolated from eucalyptus, and not surprisingly, the genotypes identified on the trees were similar to those isolated from humans in the same geographical site [5,7]. Although *C. gattii* occurs in endemic areas in Australia, the most common genotype isolated everywhere in the world is VGI [7].

*C. gattii* is typically found in tropical and subtropical climates [8]. Its recent appearance in the temperate climate region of the Pacific Northwest is cause for concern [3]. The *C. gattii* species complex has been categorized by several molecular types of the organism, VGI, VGII VGIIa, VGIIb, VGIIc, and VGIII. In Canada, where the Pacific Northwest outbreak started in 2000, the most common genotype isolated was VGII [6,9]. The infections in British Columbia in the 2000s affected humans, domestic animals, and wildlife. All the isolates of *C. gattii* from human patients in Washington belong to the VGIIa genotype. More recently, cases of *C. gattii* have been identified as belonging to variations of the VGII phenotype, specifically VGIIc [7].

*C. gattii* is commonly manifested as pneumonia and is seldom associated with infection of the central nervous system. Recently, the five *C. gattii* genotypes, VGI, VGII VGIII, VGIV, and VGVI, were recognized as five individual species, *C. gattii*, *C. deuterogattii*, *C. bacilliporus*, *C. tetragattii*, and *C. decegattii*, respectively [10]. VGII was not only the cause of the Pacific Northwest outbreak but also has a high recombination frequency via sexual macroevolution and asexual microevolution, producing the highly clonal lineages VGIIa, VGIIb, VGIIc, and VGIIx, which were associated with the dissemination of the outbreak.

Non-migratory mammals serve as sentinels for disease spread, particularly because the isolation of *C. gattii* from the environment is difficult, and animal carriers may become the sources of animal and human disease. In this report, we review the cases associated with several years of the outbreak of the disease in the State of Oregon. The diagnostic laboratory is in fact used by the Department of Public Health, and the majority of the cases of *C. gattii* diagnosed in the state were identified in the laboratory. The review of the cases in this retrospective study points to the possible continuous presence of the pathogen in the State of Oregon, and will help to establish a surveillance plan in Oregon. In this report, we review and describe several years of diagnosed *C. gattii* infections from within Oregon.

## 2. Methods and Materials

**Animals:** Materials were obtained from symptomatic animals or from necropsy specimens and cultured as described below.

### 2.1. Cryptococcus gattii Isolation and Identification

Specimens were plated onto 5% sheep blood agar (Remel, Kansas City, KA, USA) and brain heart infusion broth (Hardy Diagnostics, Santa Maria, CA, USA) and incubated at 35 °C at 6% CO_2_, as well as on Sabouraud Dextrose agar (Remel) and Mycobiotic agar (Hardy Diagnostics) and incubated at room temperature for a minimum of 7 days. All species were plated onto all the media. For the majority of cases, there was a dark gray suspension; mucoid colonies on blood agar or mucoid, opaque, white colonies on Sabouraud Dextrose agar were isolated. India ink (Hardy Diagnostics) staining was performed to verify the presence of a capsule and typical yeast morphology. Identification as *Cryptococcus* was performed utilizing Vitek MS MALDI-TOF (Biomerieux, Marcy-l’Étoile, France) and/or traditional biochemical methods. We observed hydrolysis of Christiansen’s urea (Remel), blastoconidia observed on Cornmeal agar (Remel), and brown pigment on Birdseed agar (Remel).

Differentiation between *Cryptococcus gattii* and *Cryptococcus neoformans* was achieved with Canavanine-glycine-bromothymol blue agar (Hardy Diagnostics) or MALDI-TOF. *Cryptococcus gattii* were subtyped by a reference laboratory (CDC).

**MALDI-TOF**: Isolates were identified with Vitek MS MALDI-TOF (Biomerieux). Isolates were cultured for 16–24 h and were tested from non-selective media by the manufacturer’s recommendation.

### 2.2. Subtyping of C. gattii

Isolates were identified by species and genotype in the fungal reference laboratory at the US Centers for Disease Control and Prevention. Briefly, DNA was isolated using one of several commercially available DNA extraction kits (Promega, Madison, MI, USA). Isolate subtyping was then performed following the multilocus sequence typing (MLST) scheme described by Meyer et al. [11]. The *URA5*, *IGS1*, *CAP59*, *LAC1*, *GPD1*, *PLB1*, and *SOD1* gene fragments were amplified for each isolate, and the allele numbers and sequence types (STs) were determined using the publicly available *C. gattii* MLST database (https://mlst.mycologylab.org/page/CG%20Main, accessed on 16 December 2024). Any new alleles identified in the study were submitted to the database for new allele number assignment.

### 2.3. Oregon Climate Data Methods

Climate data for Oregon from 2008 to 2022 were obtained from NOAA’s open data portal at the National Centers for Environmental Information (NCEI) (https://www.ncei.noaa.gov/cdo-web/, accessed on 16 December 2024). The search tool is then accessed underneath “Discover Data by”. The Global Summary of the month is entered into the field “selected weather observation type/dataset”. The data ranges were obtained in 6-month intervals from 2008 to 2022. Climate changes at all NOAA stations in Oregon were included for each 6-month interval of data retrieval. Monthly summaries were sourced from the Global Historical Climatology Network daily database and accessed through the climate data online interface managed by the NOAA NCEI.

Under selected weather observation data type/dataset, daily summaries were selected. The database description can be found at https://wwc.ncei.noaa.gov/pub/data/cdo/documentation/CHCND_documentation.pdf (accessed on 16 December 2024).

Wildfire occurrence data for the same period were obtained from the Oregon Department of Forestry through the Oregon.gov open data portal.

## 3. Results

### 3.1. Incidence of Infection by Counties and Month of the Year

When looking at the months of the year in which the infection was identified to determine whether there was a seasonal association with the infections caused by *C. gattii*, we observed no clear evidence of it, although 35.1% of the cases were diagnosed during the Winter months and 25% were identified during the Spring (Table 1).

### 3.2. Cases Identified by County from 2008 to 2019

Among the Oregon counties, Marion, Clackamas, Benton, and Lane were the places in which the infection was most frequently diagnosed (Table 2). Cases began to disappear in 2016, although counties such as Lane continued to report some cases after this date.

### 3.3. Genotypes/Clonal Lineage Isolated by Counties

When correlating the infections by counties with the *C. gattii* serotype and clonal lineage, we can see that Lane County is different, since the infections were caused by a great array of serotypes; in Marion County, the serotypes VGIIa and VGIIc were the most common. In contrast, in the remaining counties, the serotype VGIIa was definitively the most frequently isolated disease-causing agent in animals (Table 3).

### 3.4. Serotypes Isolates Between the Years 2008 and 2019

The most frequent serotypes and clonal lineage isolated from animals for the entire period of the study belong to groups VGIIa and VGIIc, with 63 and 23 cases, respectively, out of a total of 109 cases (Table 4). Cases associated with different serotypes and clonal lineage were also observed during the period; the serotype VGIIb was isolated from 12 cases, with 11 of the cases diagnosed from 2012 to 2015.

### 3.5. Genotype/Clonal Lineage Isolated per Animal Species

In the analysis of the species affected by different *C. gattii* serotypes and clonal lineages, serotype VGIIa was the most commonly isolated among felines and canines, although other serotypes were isolated from those two animal species. Serotypes VGIIc and VGIIb were also obtained from a large number of feline and canine patients. Most of the infections in camelids were caused by the serotype VGIIa as well as 100% of the infections diagnosed in ferrets (Table 5).

### 3.6. Sites of Infection in Different Animals Species

The analysis of the sites of infection in the different animal species showed that while felines and canines had *C. gattii* frequently isolated from the nasal cavity, skin, and lungs, canines had also infections associated with the oral cavity and central nervous system. Lung infections were seen in felines, caprines, equines, elk, and camelids (Table 6).

### 3.7. Investigation into the Weather Patterns

More cases of *C. gattii* were identified during the months representing the Winter and the Fall seasons, probably due to the rain. The months of January and November had the majority of the cases reported. While fires did not affect the incidence of infections (Table 1, Supplemental Material, the speed of the wind had an almost inverse correlation with the incidence of infection; in other words, in many of the Oregon counties, the occurrence of infection happened when the wind was less intense, between 1.8 and 3.9 miles per hour compared to greater than 4.0 miles per hour, with standard deviation not greater than 0.8 (*p* < 0.03 comparing the sites with greater incidence to the others with smaller incidence). The regions with more wind had a large variation in wind speed. The east of the state reported far less cases then the more populated west counties.

## 4. Discussion

*Cryptococcus gattii* was first described by Gattii as a distinct strain from *Cryptococcus neoformans* in 1970 in a Congolese boy, and was primarily considered a tropical disease in the 1980s [8]. It was promoted to species status later [12]. Despite being similar to *Cryptococcus neoformans* in many aspects, it is now clear that many features of *C. gattii* are very distinct. In addition, many of the known *C. neoformans* virulence factors have not been studied in *C. gattii* [13].

The main endemic areas in the world for *C. gattii* infections are the Pacific Northwest of North America (where *C. deuterogattii* is the predominant species) and California (most common found species are *C. bacillisporus* and *C gattii*), South America (Brazil and Colombia), Australia, Southern Africa, and India [14]. The clinical signs of the disease in people depend on the site of infection. In cats, nasal discharge and sneezing as respiratory manifestations and sudden blindness and seizures are usually found (CNS findings), while in dogs, lesions in the nasal cavity, granulomas in the lungs, meningoencephalitis, and eye manifestations are the common findings. In horses, the most described findings are obstructive masses in the nose [15].

Cryptococcal disease in animals has often been characterized by compromise of the respiratory system, subcutaneous nodules, central nervous system (CNS) disorder, and ocular disease [3,16]. Initially, the infection was described in British Columbia and Washington State, with *C. gattii* VGIIa as the only identified serotype and genotype [1,2]. The infection had been suggested to be linked to soil disturbance and the kind of trees predominant in the Pacific Northwest [6,12], although many questions remain.

In this study, we analyzed all the cases reported to the Oregon Public Health system from 2008 to 2019. *C. gattii* VGIIa was the most common genotype identified but may other genotypes were also isolated from infected animals. The genotypes VGIIa and VGIIb were involved in the outbreak reported from Vancouver Island in 2003, but the data reported in the current study show that additional genotypes have been isolated from animals in Oregon. Because both wild animals and domestic animals can migrate or be relocated, the introduction of different genotypes can occur. It is of interest, however, that all the cases identified in elk were secondary to *C. gattii* VGIIa infection, which match with the wildlife cases identified in 2003. The great majority of cases caused by genotypes other than VGIIa or VGIIb in Oregon were seen in canines and felines.

Lane and Marion Counties had the bulk of genotype VGIIc cases, while the cases caused by *C. gattii* VGIIa were widespread among the diverse counties. It is interesting that in Oregon, the two more predominant genotypes of *C. gattii* were VGIIa and VGIIc, which diverge from the origin of the epidemic [17]. In fact, during the last four years, the number of cases caused by the genotype VGIIc surpassed the cases caused by the genotype VGIIa. The cause of the recent predominance of the phenotype VGIIc is unknown, but curiously, the great majority of infections caused by the genotype (19 out of 23 cases) were diagnosed either in dogs or domestic cats. If there is any advantage for the genotype VGIIc to infect or survive in dogs and cats, it is currently unknown.

Both *C. neoformans* and *C. gattii* are known to cause infection in the lung and in the central nervous system [12,16,18]. In our limited collection of cases, no respiratory infection was diagnosed in dogs, in spite of the fact that it is a very common site of infection in all other animal species. Dogs, domestic cats, and camelids were observed with *C. gattii* infection in the central nervous system, which were not seen in other species. Skin infection was only identified in canine, feline, and caprine species, which may raise some questions about the environmental sources of infection. If considering the season of the year and sources of exposure, the Summer months or the Fall should be the most frequent months associated with the infection, which was not the case, since the Winter months were the months when the diagnosis was more likely. A possible explanation for this observation would be that the infections may have been acquired during the Fall but only manifested and diagnosed in the Winter, since the incubation period is supposed to vary between 6 weeks and 13 months. Ecological observation points to the increase in infection during the windy months in the Fall, which would add support to the hypothesis, although areas with more cases reported were apparently associated with lower wind speeds than the other areas in the state. Future work will be needed to address the issue, with the suggestion that additional factors may be involved [18].

It is interesting to point out that the genotypes identified more commonly, both in humans and in animals, in the outbreak in the Pacific Northwest, VGIIa and VGIIc, are very virulent [19], with the genotype VGIIc probably evolving from VGIIa because of the mating conditions in the region [20]. Of note, studies carried out at the beginning of the year 2000 showed that *C. gattii* completes its sexual cycle in association with plants [20].

An expected observation is that *C. gattii* infections were more commonly detected in counties with greater human populations, which is where most of the domestic cats and dogs live. The cases peaked between 2009 and 2014, but a small number of animals have since been diagnosed with the disease after this period. Among the counties in Oregon, Lane, Marion, and Benton are the ones with the majority of cases during the last 4 years.

Regarding the treatment for the infection, recent studies have shown that the susceptibility to azoles tends to be diminished within the *C. gattii* species complex, mainly with strains belonging to the genotype *C. deuterogatti*, when compared to *C. neoformans* [21], although others have not observed a difference [4].

Some limitations of the study are the cases diagnosed but not necessarily included in the analyses, as well all the cases lost in the forests. The study may also be biased in terms of manifestations of the disease due to lost cases. These are common difficulties of epidemiologic studies and studies which involve wildlife.

In summary, we described the epidemiologic analyses of the cases of *C. gattii* in the State of Oregon from 2008 to 2019. The infection has become endemic in the state, but our data reveal that the peak thus far has ablated, and just a few cases have been identified annually. There is some evidence that *C. gattii* has increased resistance to azoles, which can develop into a public health issue if the epidemic re-emerges.

## 5. Conclusions

This work assembled the cases of animal infection caused by *C. gattii* diagnosed in Oregon during the outbreak of 2008–2019. *C. gattii* VGII (*Cryptococcus deuterogatti*), with the genotypes VGIIa, VGIIb, and VGIIc, was responsible for 98% of the cases. VGIIa was identified in more than 50% of the animals, and *Cryptococcus bacilliporus* (VGIII) was only isolated from cat patients. The most common site of infection in dogs was the brain; in cats, this was the nasal cavity and the skin, while the lung was the most affected site in caprines, equines, camelids, and elk. Marion and Lane Counties account for the majority of the infections, followed by Clackamas, Benton, and Multnomah Counties. The infection was predominantly identified during the Fall and Winter months, except for Benton County, where it was seen more commonly during the Summer months. The reasons for the outbreak that lasted for many years is still unknown. This work will facilitate the identification of the infection in case of another outbreak and the possible transmission between humans and animals.

## Figures and Tables

**Table 1:** Incidence of diagnosed infection by counties and months of the year.

Counties	Months of the Year											
	1	2	3	4	5	6	7	8	9	10	11	12
Marion	3	5	2		1	4	1		2	2	1	
Multnomah	5		1		1	2					2	2
Lincoln		1	1		1			1		1		
Columbia	1											
Clatsop			1									
Clackamas		2				1	2	1	4	1	3	1
Benton			1	1		3	6		1		1	1
Polk				1			1			1		
Washington	2	1		1	1					1	2	
Lane	6	2		1	1	2				1	5	1
Yamhill	2	1		1	1							
Josephine			1									1
Linn								1				
Deschutes										1		
Total	18	11	8	4	6	13	10	3	7	8	14	6
Season	38 (Jan.–Mar.)			23 (Apr.–June)			20 (Jul.–Sep.)			28 (Oct.–Dec.)		

**Table 2:** Cases identified in counties from 2008 to 2019.

Counties/Year	08	09	10	11	12	13	14	15	16	17	18	19
Marion	2	1	3	5	5	0	1	4	3	0	0	0
Multnomah	2	0	4	3	1	0	1	1	1	0	0	0
Lincoln	1	0	2	0	0	1	0	0	1	0	0	0
Columbia	0	1	0	0	0	0	0	0	0	0	0	0
Clatsop	0	1	0	0	0	0	0	0	0	0	0	0
Clackamas	0	4	0	2	4	0	1	0	1	0	0	1
Benton	0	1	0	5	1	1	1	0	3	0	0	1
Polk	0	2	0	0	0	0	1	0	0	0	0	0
Washington	0	0	1	0	0	3	4	0	0	0	0	0
Lane	0	0	2	3	0	6	3	1	0	3	0	1
Yamhill	0	0	0	1	0	2	0	1	1	0	0	0
Josephine	0	0	0	1	0	0	0	1	0	0	0	0
Linn	0	0	0	0	1	0	0	0	0	0	0	0
Deschutes	0	0	0	0	0	0	1	0	0	0	0	0
Total	5	10	12	20	12	13	13	8	10	3	0	3

**Table 3:** Genotypes isolated by counties.

County	Total	VG I	VG II	VG IIa	VG IIb	VG IIc	VG III
Marion	22	1		9	2	10	
Multnomah	11			9		1	1
Lincoln	5			5			
Columbia	1				1		
Clatsop	1					1	
Clackamas	14		1	10		2	1
Benton	14	1		9		3	1
Polk	3			2		1	
Washington	9	1		6	2		
Lane	22		3	7	6	5	1
Yamhill	5			3	2		
Josephine	2			2			
Linn	2			2			
Deschutes	1				1		
Total	109	3	4	63	12	23	4

**Table 4:** Isolated serotype clonal lineages causing disease over the years.

Year/Genotypes	VGI	VGII	VGIIa	VGIIb	VGIIc	VGIII	Yearly Total
2008			4		1		5
2009			6	1	2		9
2010			12				12
2011	1		14		3	2	20
2012			7	2	2		11
2013	1	3	7	2	1	1	15
2014	1		6	5	1		13
2015			2	2	3	1	8
2016			4		6		10
2017			1		2		3
2018							0
2019		1			2		3
Total	3	4	63	12	23	4	109

**Table 5:** *Cryptococcus gattii* genotypes isolated from the different species of animals.

Genotypes	VGI	VGII	VGIIa	VGIIb	VGIIc	VGIII
Canine	1		20	3	6	
Feline	2	1	19	7	11	4
Caprine		2	7		2	11
Equine		1		1		3
Elk			4	1		
Camelid			10		1	
Ovine					1	
Ferret			4			
Total	3	4	63	12	23	4

**Table 6:** Sites of infection for identification of *C. gattii*.

Sites	Canine	Feline	Caprine	Equine	Elk	Camelid	Ovine/Others
Lung	1	5	6	2	3	7	
Oral/gum Tooth abscess	5	3					
Kidney		2					
Brain/ Spinal Fluid	9	4				4	
Nasal cavity	3	15	1		1		
Granuloma		3					
Skin	5	12	3				1
Liver						1	
Sinus Swab	2	1				1	
Eye	1	1					
Sub-Mandibular Node	4	3					
Mass	1	3	1		1		
Bone	1						

## Data Availability

All the available data are included in the manuscript. Additional information can be obtained from the corresponding author.

## Supplementary Materials

The following supporting information can be downloaded at https://www.mdpi.com/article/10.3390/vetsci12020185/s1, Excel S1.
